# Supplementary material for: ‘It’s like being in a war with an invisible enemy’: A document analysis of bereavement due to COVID-19 in UK newspapers
Source: PLoS One. 2021 Mar 4;16(3):e0247904. doi: 10.1371/journal.pone.0247904 (PMC7932501; doi:10.1371/journal.pone.0247904)
Supplement: S1 Table — (DOCX) [file pone.0247904.s001.docx]

**S1 Table. Number of included articles by date, contextualised with total deaths to date and key events**

| **Week** | **Date** | **Number of articles** | **Total cumulative number of deaths recorded (from GOV.uk^48^)** | **Key events** |
| --- | --- | --- | --- | --- |
|  | 11/01/2020 |  |  | China reports first death from COVID-19^1^ |
|  | 29/01/2020 |  |  | 1st positive test for COVID-19 in the UK |
|  | 06/02/2020 |  |  | First COVID-19 death in the USA (confirmed via autopsy)^2^ |
|  | 28/02/2020 |  |  | First UK citizen death from COVID-19 abroad |
|  | 05/03/2020 |  |  | First confirmed hospital death from COVID-19 |
|  | 06/03/2020 |  |  | First confirmed care home death from COVID-19 |
|  | 11/03/2020 |  |  | World Health Organization declares COVID-19 a pandemic |
|  | 12/03/2020 |  |  | Chief medical officer raises UK threat from moderate to high |
|  | 14/03/2020 |  |  | UK government's “herd immunity” strategy widely criticized |
| **1 (A)** | 18/03/2020 | 2 | 115 | Church of England issues funeral guidance |
|  | 19/03/2020 | 4 | 158 |  |
|  | 20/03/2020 | 1 | 194 | Schools, restaurants, pubs, theatres and cinemas close; nightclubs, theatres, cinemas, gyms and leisure centres are instructed to close |
|  | 21/03/2020 | 2 | 250 |  |
|  | 22/03/2020 | 1 | 285 |  |
|  | 23/03/2020 | 9 | 359 | Lockdown announced |
|  | 24/03/2020 | 0 | 508 |  |
| **2** | 25/03/2020 | 1 | 695 |  |
|  | 26/03/2020 | 5 | 878 |  |
|  | 27/03/2020 | 2 | 1162 |  |
|  | 28/03/2020 | 0 | 1456 | First “clap for carers” – continued every Thursday until the 28th May |
|  | 29/03/2020 | 5 | 1670 | 1st reported death of a clinician in the UK |
|  | 30/03/2020 | 2 | 2044 |  |
|  | 31/03/2020 | 0 | 2426 | Public Health England publishes new guidance for safe funerals following discussions with faith leaders; Office of National Statistics begins to include non-hospital deaths in daily statistics |
| **3** | 01/04/2020 | 0 | 3096 | 1st recorded child death in the UK |
|  | 02/04/2020 | 2 | 3748 |  |
|  | 03/04/2020 | 8 | 4462 | The Sun launches an appeal to raise £1,000,000 for NHS staff |
|  | 04/04/2020 | 4 | 5222 |  |
|  | 05/04/2020 | 8 | 5866 | Queen's speech addressing nation about coronavirus |
|  | 06/04/2020 | 4 | 6434 |  |
|  | 07/04/2020 | 0 | 7472 |  |
| **4 (B)** | 08/04/2020 | 3 | 8506 |  |
|  | 09/04/2020 | 5 | 9616 |  |
|  | 10/04/2020 | 4 | 10768 | Health Secretary Matt Hancock warns clinicians not to overuse Personal Protective Equipment |
|  | 11/04/2020 | 5 | 11608 |  |
|  | 12/04/2020 | 9 | 12294 |  |
|  | 13/04/2020 | 8 | 13038 |  |
|  | 14/04/2020 | 2 | 14085 |  |
|  | 15/04/2020 |  |  | Matt Hancock’s statement on increasing visits for people at the end of their lives |
|  | 16/04/2020 |  |  | UK lockdown extended by 3 more weeks |
|  | 23/4/2020 |  |  | NHS Confederation publishes member briefing *The impact of COVID-19 on BME (Black and minority ethnic) communities and health and care staff* |

^1^ <http://www.xinhuanet.com/2020-01/11/c_1125448269.htm>

^2^ <https://edition.cnn.com/2020/04/23/us/california-woman-first-coronavirus-death/index.html>
